# Supplementary material for: Are carbon emissions trading and green financial instruments synergistic? -Comprehensive quantitative research based on content analysis
Source: PLoS One. 2024 Mar 7;19(3):e0298601. doi: 10.1371/journal.pone.0298601 (PMC10919601; doi:10.1371/journal.pone.0298601)
Supplement: S1 Data — (ZIP) [file pone.0298601.s001.zip › Data/地级市Z_F工作报告.zip!/╒π╜¡/╜≡╗¬╩╨/└┤╫╘╣1⁄2╓┌║┼í╛┬φ┐╦╩2╛▌═°í┐_2014.docx]

各位代表：

现在，我代表金华市人民政府向大会报告工作，请予审议，并请市政协委员和其他列席人员提出意见。

一、2013年工作回顾

　　过去的一年，市人民政府在中共金华市委领导下，全面贯彻党的十八大和十八届二中、三中全会精神，坚持“百姓富裕、浙中崛起”总目标，着力稳增长调结构，抓治水促转型，办实事惠民生，正作风提效能，与全市人民一道齐心协力，攻坚克难，较好地完成了市六届人大四次会议确定的主要目标任务。全市实现生产总值2959亿元，增长9.1%；地方财政收入242.5亿元，增长12.8%；城镇居民人均可支配收入36423元，增长9.8%；农村居民人均纯收入14788元，增长11.3%。一年来，我们主要做了七方面工作：

（一）保持经济平稳发展。抓项目扩大投资。加快推进重大项目建设，开展克难攻坚活动，项目实施进度明显加快。固定资产投资1364亿元，增长21.1%。省“411”重大项目、市重点项目分别完成年度投资计划的169%和156%。创新招商选资方式，注重招大引强，浙（婺）商回归项目309个，到位资金157亿元，新引进世界500强企业4家。抓市场扩大消费。开展拓市场促消费活动，举办义乌小商品博览会、永康五金博览会、国际商贸发展大会、浙中购物节等展会。提升实体市场，发展网上市场，积极培育消费热点，社会消费品零售总额1407亿元，增长14.8%。商品市场成交额2121亿元，增长11.4%。抓改革扩大出口。发挥义乌国际贸易综合改革试点效应，带动外贸出口增长，实现外贸出口总额325亿美元，增长52.7%。坚持稳增长与调结构相结合，实体经济平稳发展。粮食生产保持稳定，工业效益逐步回升，建筑业加快发展，房地产业稳定发展，地方财政收入增长结构进一步优化。金融机构本外币存款余额6161.6亿元，贷款余额5157.3亿元，分别增长15.7%和18.7%。

（二）加快产业转型升级。强化科技创新。开展“十倍增两提高”科技服务专项行动，推进园区合作，实现中科院金华科技园与市高新技术产业园区融合发展，金华国家农业科技园区获批。新增国家级高新技术企业46家、省级重点企业研究院3家、省重点产业技术联盟4家。新增省级以上科技项目477个，高新技术产业增加值占规上工业增加值比重达到20.6%。实施“双龙计划”，入选国家“****”5人、省“****”17人，新增院士工作站5个。推进产业集聚平台建设。完成金华经济技术开发区和金西经济开发区整合工作。启动永康现代农业装备高新区、浦江水晶产业生态集聚区等建设。加快培育11个省级服务业集聚示范区，横店影视文化产业实验区列为国家级文化和科技融合示范基地。加大农业“两区”建设力度，新增省级现代农业综合区2个、特色农业精品园12个。实施“四换三名”工程。改造提升传统产业，培育发展新兴产业，永康“机器换人”、“空间换地”等经验在全省推广。华东铝业实现战略重组。实施“亩产倍增”行动计划，加大旧厂区改造和闲置低效用地处置力度，消化批而未供土地26055亩，盘活存量建设用地7956亩。大力发展网络经济，我市入选首批信息消费国家试点城市、全国十大电子商务城市，电子商务交易额1816亿元、网络零售额668亿元，分别增长90%和123%。推进质量和品牌强市建设，新增中国驰名商标9件。培育壮大市场主体。完成“个转企”13102家，净增“小升规”企业938家、“下升上”企业403家，新增股份制公司13家，新增中国民营企业500强2家。新增家庭农场348家，规范化合作社达到592家，省级以上农业龙头企业达到39家。

（三）大力推进城乡建设。加快编制城市群专项规划。完成浙中城市群综合交通、水资源配置等专项规划，编制城市地下空间开发利用规划，完善城镇规划体系。加快重点功能区块建设。统筹推进多湖、湖海塘、五百滩、尖峰山、西客站及二七新村、古子城、旅游度假区等区块建设，完善开发机制，优化功能定位，开展土地征迁、招商选资和项目前期工作。“三江六岸”景观整治提升工程有序推进。加快金义都市新区六大区块开发和十条主干道建设，菜鸟·金义电子商务新城等重大产业项目顺利开工。加强中心镇中心村建设。出台加快推进中心镇发展改革暨小城市培育若干意见，深化横店镇和佛堂镇小城市培育，中心镇建设取得新成效。澧浦镇获评浙江最美乡镇，花园等3个村获评浙江最美乡村，建成12条美丽乡村风景线。加快基础设施建设。推进杭长客专、金温铁路扩能改造、东永高速公路等项目建设，基本建成十白线、怀万线二期等公路，扎实做好金台铁路、临金高速公路等项目前期工作。启动衢江金华段航运开发项目。全力支持两项国家级特高压工程建设。稳步推进金丽温输气管道工程。加强农田水利基础设施建设，完成钱塘江干堤等堤防加固60公里。加强城市管理。推进精品城市建设，成功创建国家园林城市，国家卫生城市通过复审，市区和磐安成功创建省级森林城市。调整完善了市区城市管理行政执法体制。

（四）加大重点改革力度。认真抓好义乌国际贸易综合改革试点。市场采购贸易方式获批试行，全面实施主体准入、货物通关、财税外汇等贸易便利化措施，成功创建国际陆港城市。加快低丘缓坡综合开发利用试点步伐。累计完成农转用征收报批面积4.7万亩，完成率达99%，全面落实试点区耕地占补平衡。有序推进商务部四项改革试点和国家智慧城市、省级智慧车联网等省部级改革试点。深化行政审批制度改革。实行政府投资项目并联审批和行政审批事项目录化管理，市级行政许可事项由375项减少为189项，非行政许可审批事项由231项减少为97项，审批平均承诺期限由7个工作日减少为5个工作日。开展中介机构和社会团体清理整顿等专项执法监察。

（五）加强环境综合整治。全力推进水环境治理。全面实行“河长制”，强势推进浦阳江、金华江等重点流域水环境综合治理。加强重污染高耗能行业整治，关停淘汰电镀、印染、造纸、化工等企业84家。浦江关停、取缔水晶加工户1.4万家，淘汰水晶设备7.2万台，治水经验在全省推广。抓好农业面源污染整治，拆除养殖场3608家，新建畜禽养殖场治污设施285家。整治制沙场（点）537家，兰溪制沙整治取得明显成效。加快建设城镇生活污水处理设施，建设城镇污水配套管网377公里，开工建设秋滨污水处理厂二期工程。推进农村生活污水治理，行政村覆盖率达72%。市区联合执法，加强沙金兰、九峰等水源涵养生态功能区污染整治和生态保护，关停拆除养殖场95家、锯板厂49家，生态涵养区面积扩大19.5万亩。大力推进“三改一拆”。拆除违法建筑1490万平方米，完成旧厂区、旧住宅区、“城中村”改造802万平方米。坚持政府主导、市区联动，统一政策、规划引导，加快推进市区二环路以内“城中村”改造试点，开工建设横塘沿“城中村”改造安置房。积极推进交通治堵。市区建设双向公交专用道11.2公里，新增公交车60辆，公交分担率提高到16%。新增停车位1.3万个。改造城市道路10条，打通断头路4条，建设智能交通控制系统，探索“十字路口”精细化、人性化管理，道路通畅和安全水平得到提高。投放公共自行车5100辆，其中市区投放3000辆。制定落实大气复合污染防治实施方案，市区淘汰黄标车5532辆。统筹推进“四边三化”、“双清”行动、“百村示范、千村整治”工作。武义加快推进生态景区全域化。

（六）重视发展民生事业。着力保障和改善民生。财政民生支出占财政总支出的比重达到75.1%。加强就业和社会保障工作，新增城镇就业7.1万人，城乡居民社会养老保险参保率达97%，医疗保险参保率达96%。市区开展大病医疗商业补充保险工作，完善被征地农民基本生活保障机制。积极推广金东新型农村居家养老模式，新建成农村社区居家养老服务照料中心537个、省级示范农村社区服务中心66个。新开工建设保障性住房7203套，基本建成6105套，实施农房改造22033户，农村困难家庭危房改造2386户。不断扩大社会救助覆盖面，城乡低保标准提高10%以上，困难群众基本生活价格补贴7.6万人次，救助困难群众2.1万人次。扎实开展新一轮扶贫工作，列入省级扶贫重点村576个，山区群众异地搬迁完成5210人，婺城、磐安等下山移民新村建设进展顺利。

认真抓好各项社会事业。创建义务教育标准化学校40所、省一级中等职业学校10所、省特色示范普高5所、省等级幼儿园226所，市特殊教育中心开工建设。启动市级公立医院改革，完善基层医疗卫生机构绩效考核体系。加快推进市中心医院东扩工程，市中医院新病房楼、兰溪人民医院城西院区、东阳人民医院综合大楼投入使用。完善公共文化基础设施和服务体系，中国婺剧院、市文化艺术中心对外开放，建成农村文化礼堂218家，长乐等8个村列为省级历史文化村落保护利用重点村。市体育中心投入使用，坚持节俭办会，成功举办省第二届体育大会。稳步推进人口计生工作，全面发展老龄、慈善、残疾人和关心下一代等事业。创新基层社会治理方式。开通8890便民服务平台，试运行以来日均受理市民诉求事项293件，累计办结18475件，办结率97%，满意率98%以上。进一步完善信访工作机制，加强矛盾纠纷调解和社区矫正工作，深入开展“六五”普法。推进食品药品安全监管长效管理，保持安全生产严管高压态势。深入推进“平安金华”建设，连续七年获浙江省“平安市”称号，连续五年跻身全国30个最安全城市，社会保持和谐稳定。支持工会、共青团、妇联等人民团体开展工作，加强社工专业人才和志愿者队伍建设。加强民族宗教、侨务、对台、应急、气象、民防、统计、档案、地方志、仲裁等工作。积极开展国防动员、双拥和优抚安置工作。深入推进援疆、援藏、援青及“双对口”工作。

（七）加强政府自身建设。坚决贯彻落实中央“八项规定”、省委“28条办法”和“六条禁令”，按照市委部署，扎实开展“正风提效促赶超”专项活动。发挥市民问政、行风热线、市长信箱等平台作用，推进服务型政府建设。认真落实人大决议，自觉接受人大法律监督和工作监督、政协民主监督和群众社会监督，扎实办理人大代表建议298件、政协提案502件。加强规范性文件审查和行政复议工作。加强审计监督和行政监督，完善廉政风险防控机制。加强公务接待、公务用车、因公出国（境）管理，“三公”经费支出同比下降14.4%。

各位代表，过去一年我市取得的成绩来之不易。这是省委省政府和市委正确领导的结果，是全市人民共同努力的结果。在此，我代表市人民政府，向全市人民，向市人大代表、市政协委员，向离退休老干部，向各民主党派、工商联、人民团体和社会各界人士，向驻金解放军和武警部队官兵，向中央和省驻金单位，向所有关心支持金华发展的朋友们，表示崇高的敬意和衷心的感谢！

同时，我们也清醒地看到，我市城镇居民人均可支配收入增速没有完成预期目标；十方面50项民生实事中，由于国家政策调整，义务教育发展基本均衡县（市、区）评估验收延迟，没有按时完成；创新驱动和转型升级的基础还不够稳固；一些重点领域和关键环节改革还不够到位；中心城市功能还需进一步提升；教育、医疗、环境治理等工作与群众期待仍有较大差距，公共服务和社会治理能力亟待提高；政府职能转变相对滞后，作风建设有待加强，少数干部服务能力、工作效能和廉洁意识还需进一步提高。对上述问题和不足，我们一定高度重视，切实加以解决。

　二、2014年主要工作安排

　　2014年是全面深化改革的开局之年。政府工作的总体要求是：全面贯彻党的十八大和十八届二中、三中全会精神，认真落实省委省政府及市委的决策部署，深入实施“八八战略”，紧紧围绕“百姓富裕、浙中崛起”总目标，以改革统领全局，坚持稳中求进、改中求活、转中求好，以“五水共治”为突破口，全面实施“八项行动”，着力转变政府职能，着力强化环境综合整治，着力加快转型升级，着力推进城乡一体化，着力保障改善民生，为推进赶超发展，建设“两富”现代化都市区奠定更加坚实的基础。

建议2014年全市经济社会发展的主要预期目标是：地区生产总值增长8.5%，地方财政收入增长8.5%，城镇居民人均可支配收入增长9.5%，农村居民人均纯收入增长10%，节能减排指标完成省下达的目标任务。

（一）实施“改革创新”行动，增强赶超发展内生动力

开展“四破”专项整治活动，建立健全“四破”体制机制，推进资源要素集约节约利用和公共资源集中管理、市场化运作。建立以企业亩产效益为核心的综合评价体系，出台闲置低效用地再开发利用政策，推进水电气、建设用地、环境容量等资源要素差别化定价，建立健全工业用地、节能量、水权、排污权等交易制度，完善资源有偿使用机制，推进环境污染第三方治理。优化民营经济发展体制机制，进一步落实扶持发展民营经济的各项政策，引导民间资本投向基础设施、基础产业和社会事业等领域。加快金融改革创新，完善金融服务体系，破解实体经济面临的资金要素供给困难。支持东阳、永康开展民间资金管理公司、借贷登记服务中心试点。加强国有资产运行管理，推进国有投融资平台建设。积极发展混合所有制经济。

落实义乌国际贸易综合改革试点第二个三年实施计划，对接上海自由贸易区，深化国土、金融等专项改革，完善国际贸易管理体系，促进贸易便利化。扎实推进低丘缓坡综合开发利用试点，加快征地拆迁和基础设施建设，同步推进招商选资和产业集聚区建设。积极争取废弃工矿地复垦利用试点。

全面实施创新驱动发展战略，力争研究与试验发展经费支出占生产总值比例达到1.95%。整合提升国家级、省级开发区（园区），大力推进金华经济技术开发区新能源汽车产业园、现代生物医药产业园、电子信息产业园、文化创意产业园建设。以中科院金华科技园与市高新技术产业园区整合为契机，推进高科技产业园建设。加快永康现代农业装备高新区、浦江水晶产业生态集聚区等产业平台建设，创建国家级文化产业示范园区，推进东阳文化产业全域化发展。发挥企业创新主体作用，引导民营资本与人才智力项目对接，促进创新资源向企业集聚，争创国家级企业技术中心，新增高新技术企业100家。完善产学研结合机制，办好工科会，推动校企合作和协同创新，鼓励设立院士专家工作站、企业研究院，加快建设浙江高校产学研联盟金华中心、大学院所金华技术转移中心。大力实施“****”、“双龙计划”，引进一批创新团队和领军人才。加强企业家队伍建设，加大本地人才培养力度。

（二）实施“美丽金华”行动，强化环境综合整治

大力推进“五水共治”。认真落实市人大常委会关于加强金华江流域水环境综合整治的决定，依靠全市人民，全面实施金华江、浦阳江等流域水环境综合治理。以重整山河的雄心和壮士断腕的决心，打响“保护母亲河”攻坚战。按照“一年灭黑臭”的要求，全面整治垃圾河、黑河和臭河。全面开展“治砂清水”专项行动，建立砂石资源管理长效机制。继续实施重污染高耗能行业整治。抓好农村面源污染治理，严格执行畜禽养殖禁养区、限养区制度，加强养殖污染物无害化处理和资源化利用。全面编制雨污分流管网建设规划，加快实施城市污水处理厂扩容、城乡截污纳管、集镇污水处理设施改造等重点治污工程，市区污水处理率达到90%以上，县城污水处理率达到85%以上，建制镇污水处理率达到40%以上。开展最美河流示范工程建设。进一步扩大水源涵养生态功能区范围，加快库区群众下山生产生活步伐，创新功能区水源保护管理体制机制，保护好全市人民的“大水缸”。开工建设安地水厂，推进武义石硖水库、磐安花溪水库、永康北部水库联网等重点供水工程建设，统筹城市群水资源配置。加快城市堤防加固改造，完成干堤加固和中小河流治理110公里，病险水库除险加固29座。修编市区防洪排涝规划。实施灌区节水配套改造工程，探索中水回用和污水再生利用，创建节水型城市。

深入实施“三改一拆”。坚持应拆尽拆、拆改建管并举，坚决遏制新违建，努力消除存量违建，开展无违建县、乡、村创建活动。实施“城中村”改造三年行动计划，全市“城中村”改造面积600万平方米，其中，市区推进15个“城中村”改造项目，开工安置房85万平方米，各县（市）实施3-5个“城中村”改造项目。推进旧住宅区改造，启动背街小巷改善三年行动计划，开展环境整治，改善基础设施、街景立面和功能配套，改造旧住宅区230万平方米。推进旧厂区改造，加快园区“改二优二”、城区“退二进三”步伐，改造旧厂区150万平方米。

积极实施大气污染防治。制定实施雾霾天气应急处置预案，推进禁燃区建设，加快城区燃煤锅炉改造淘汰步伐，深入实施水泥、热电行业脱硝工程。加强机动车尾气污染治理，加快市机动车尾气检测中心建设。严格实行黄标车区域限行，加大高排放车辆淘汰力度。整治扬尘污染，加强挥发性有机物治理。推进大气监测网络建设。

全面开展美丽乡村建设。按照“四美三宜”的要求，深化“百村示范、千村整治”工程，加大农村基础设施建设力度，为留在农村的农民创造更好的生产生活条件。结合“三改一拆”和“无违建村”创建工作，开展农村“赤膊房”整治行动。加强农村生活污水治理，着力提高治理覆盖率。推进农村生活垃圾集中处理，提高减量化、无害化、资源化处理水平。加强历史文化古村落保护利用，推进农旅结合，打造特色乡村。

（三）实施“产业升级”行动，推动经济转型发展

加快发展现代农业。加大农业“两区”和农业科技、农田水利基础设施建设投入，提高农业综合生产能力。严守耕地保护红线，稳定粮食生产面积，确保粮食安全。做强花卉苗木、蔬菜水果、特种水产、有机茶、中药材和食用菌等特色产业。在宜养区扶持生态规模养殖场建设，实施金华“两头乌”猪产业振兴行动计划。推进“兴林富民”工程。推行标准化生产，扩大无公害农产品、绿色食品、有机农产品基地面积，大力发展节水农业、循环农业、设施农业和休闲观光农业。加快金华国家农业科技园区和农业科技创新孵化中心建设，发挥浙中农业科技资源优势，建设特色优势物种研发基地，推进农业科研成果产业化。积极培育家庭农场、种养大户、专业合作社、产业化龙头企业等新型经营主体，加快推进金华农产品批发市场迁建项目，继续办好特色农业展会，构建农业专业化服务体系和农产品质量安全监管体系。发挥火腿、佛手等传统品牌优势，推动农业转型发展。

加快发展先进制造业。坚持工业强市战略不动摇，大力实施“四换三名”工程，淘汰改造高能耗、重污染企业100家，完成工业技改投资400亿元，盘活存量建设用地8000亩。促进信息化与工业化深度融合，加快传统产业改造升级，大力发展新能源汽车、电子信息、生物医药、新材料、节能环保等新兴产业。加快省级现代产业集群转型升级示范区建设，支持永康创建国家循环经济示范市（县），培育和引进一批工业设计中心。抓好新能源汽车推广应用和现代农业装备产业技术创新试点。实施大企业大集团和高成长企业培育计划，落实“个转企、小升规、规改股、股上市”扶持政策，引导金融机构加大对实体经济的支持力度，推进中小微企业健康发展。培育工业强县强镇，永康规上工业产值力争超千亿元，支持义乌、兰溪争创省级工业强县。以创建建筑业强市为抓手，做大做强建筑业。

提升服务业发展水平。大力发展电子商务、现代物流、商务会展、金融服务、高端商贸业，提升发展专业市场和传统商贸业，培育发展影视文化、设计创意、中介服务、休闲旅游养生业，健康发展房地产业，努力提高服务业在三次产业中的比重。大力发展网络经济，努力盘活房产、土地等存量资源用于发展网络经济，加快浙中信息产业园、金东信息软件创业园、市电子商务创业园扩容提升。抓好菜鸟·金义电子商务新城、金义邮政电子商务示范园等重大项目建设。开工建设金华经济技术开发区电子商务产业园项目。加快发展现代物流业，建设金义都市新区、义乌、浦江等跨境电子商务园和义乌保税物流中心（B型）。加快义乌国际生产资料市场、兰溪纺织品综合市场、永康五金城生产资料市场、武义工业材料城等建设和发展。统筹旅游资源保护和开发利用，建设一批有特色的旅游产品，促进旅游与生态、文化、会展、养生等产业有机融合，打造生态休闲旅游目的地，构建大旅游格局。

努力扩大有效需求。充分发挥有效投资对稳增长、促转型的关键作用，以“扩大有效投资月”活动为载体，抓攻坚优服务，加大项目推进和难题破解力度，实现省“411”重大项目、省重点项目全面开工建设。坚持招商选资、招大引强，引浙（婺）商、招央企、选外资，着力引进一批国内外500强企业和投资20亿元以上大项目。培育消费热点，鼓励商业模式创新，推进信息消费国家试点，优化消费环境。以开放促转型，积极发展转口贸易、进口贸易，稳定传统市场，拓展新兴市场，鼓励企业“走出去”，努力提高开放型经济发展水平。

（四）实施“群城共建”行动，构筑都市区发展新格局

科学编制都市区发展规划。以“群城聚市”战略为指导，以“一轴两带”城市组团发展为格局，以金华市区和义乌为核心，以金义都市新区为战略节点，科学编制城市群、中心城市、卫星城市、中心镇等总体规划、详细规划和各专项规划，突出城镇功能提升规划和产业升级规划的融合，充分发挥规划在推进县域经济向都市区经济转型中的龙头作用，有效引导城镇转型和发展方式转型的协同互促、同频共振，推进城乡一体化发展。

做强中心城区。加快多湖中央商务区开发，努力建设地区金融服务中心、浙中经济总部中心、金华科技文化中心和城市商务创意中心。加快推进湖海塘、五百滩、尖峰山、西客站及二七新村、古子城、旅游度假区等重点功能区块建设。加快推进“三江六岸”景观提升工程，提升项目建设的质量和文化内涵。积极推进婺城新城区、金东新城区、金华经济技术开发区金西区块建设。大力推进金义都市新区核心区建设，带动开发六大区块，加快重大基础设施和产业项目建设。加速推进义乌国际陆港物流园区、金融商务区和总部经济区建设。积极推进兰溪总部商务区、东阳红椿巷区块和永康总部中心建设，加快浦江江南新区、武义北岭新区和磐安新城区开发。

做特中心城镇。以小城市、卫星城镇、中心城镇为重点，推进扩权强镇改革，整合完善中心镇规划和开发区规划，以“五水共治”为切入点，加强城镇基础设施和主要功能区建设，培育特色产业，增强中心城镇集聚要素和承接农业转移人口就地市民化的能力。抓好佛堂、横店小城市培育试点工作，培育一批文化名镇、旅游强镇、特色小镇。

深化城乡体制机制改革。推进城乡要素平等交换和公共资源均衡配置，统筹城乡基础设施建设和社区建设。积极探索赋予农民对承包地占有、使用、收益、流转及承包经营权抵押、担保权能。深化农村股份合作制改革，赋予农民对集体资产股份占有、收益、有偿退出及抵押、担保、继承权。

加快基础设施建设。实施铁路西客站和汽车西站综合枢纽改造、金温铁路扩能改造工程，确保杭长客专金华段如期完工，启动金华铁路新货场建设，加快推进金台铁路前期工作，启动甬金铁路前期研究。加快实施杭金衢高速公路金华段拓宽和新狮互通连接线提升工程，东永高速公路建成通车，开工建设临金高速公路。加快义兰、义武公路等城际快速通道建设，实施330国道市区段及永康段、20省道浦江段、37省道义东段、40省道东磐段、42省道磐安段等改建工程，抓好金兰中线、金义中线、永康至金义都市新区公路等项目前期工作。推进义乌机场飞行区等级提升和横店通用机场建设。启动快速公交（BRT）建设。实施衢江金华段航运开发项目和兰江航道整治。续建金丽温输气管道工程。推进4G网络建设。加大电网建设改造力度，加快建设燕尾、鞋塘、汤溪等输变电工程。

提升城市管理水平。按照洁化、绿化、序化、美化的要求，推进市容市貌、环境卫生、市政设施等管理精细化。继续开展“五城同创”活动，形成人人参与创建、支持创建的良好氛围，实现人民城市人民建、人民城市人民管。继续实施城市治堵五年行动方案，治理城市主干道堵点，坚持公交优先，提高公交分担率。加快绿道慢行系统建设，提高公共自行车网络覆盖面。进一步完善智能交通系统，坚持严格管理和教育引导相结合，持续加大交通违法整治力度，提高交通参与者的文明素质。加大停车场建设和管理力度。加快智慧城市建设。

（五）实施“固本强基”行动，促进社会和谐稳定

加强基层社会治理，推行“枫桥经验”，引导群众参与社会管理，培育公益慈善类、社区服务类组织，壮大社工及志愿者队伍，实现政府治理和社会自我调节、居民自治良性互动。用群众工作统揽信访工作，把工作做在基层、做进家庭、做入心坎，把矛盾化解在基层、问题解决在当地、隐患消除在萌芽状态。落实民族宗教政策，促进民族团结、宗教和谐。

畅通市民问政、市长信箱、行风热线、来信来访等渠道，完善与群众利益密切相关的重大事项社会公示和听证制度，做好重大决策社会稳定风险评估和社会矛盾纠纷预防化解工作。深化8890便民服务平台建设，拓展服务内容，完善办理机制，努力实现市域内一张网。推进户籍管理制度改革试点，加强和创新流动人口服务管理。建立“最美金华人”道德模范关爱机制。强化司法保障，完善人民调解、行政调解、司法调解联动的工作体系，加强社区矫正工作，健全法律顾问制度。

加强食品药品质量安全监管，建立健全全过程监管和可追溯制度。建立生产安全隐患排查和预防控制长效机制，深化企业安全生产标准化建设，加强消防安全管理，有效防范较大事故、坚决遏制重特大事故发生，保持生产安全形势总体稳定。加强社会治安综合治理，完善治安防控体系，防范和打击各类违法犯罪活动。保障网络和信息安全。做好防汛防台抗旱、动植物防疫、森林防火等工作。完善应急联动管理机制，提高应急处置能力。

（六）实施“先进文化”行动，统筹社会事业发展

推进文化强市建设，开展以爱国、敬业、诚信、友善为主要内容的公民道德建设，深化文化惠民工程，加快建设农村文化礼堂，完善城乡文化设施网络，大力发展文化产业。发挥中国婺剧院、市体育中心等场馆服务功能，丰富群众文化体育生活。积极创建义务教育发展基本均衡县（市、区），推进教育现代化。实施新一轮学前教育三年行动计划，推进义务教育学校标准化建设，提高普通高中教育质量，改善特殊教育办学条件，强化职业教育实训基地和重点专业建设。支持浙江师范大学、上海财大浙江学院、金华职业技术学院、义乌工商学院等在金高校发展。推进卫生强市建设，统筹推进公立医院改革，鼓励社会资本兴办高端医疗机构，支持非营利性医疗机构发展。深化与省内外知名医疗机构、医学院校合作，提升医疗服务水平。加强基层公共卫生硬件设施和医疗队伍建设，提高乡镇（社区）公共医疗卫生服务能力。做好公共卫生、爱国卫生、精神卫生和传染病防控工作。继续加强人口计生工作，落实“单独两孩”政策，促进人口长期均衡发展。

实施积极就业政策，努力做好高校毕业生、城镇困难人员等重点群体就业工作。扎实开展社保扩面征缴工作，健全城乡居民、城镇职工养老保障机制，加快城乡医疗保障制度整合步伐，推进医保“一卡通”建设。全面推广金东新型农村居家养老模式，探索建立城市社区社会化养老模式。加快推进保障性住房建设。加强残疾人等城乡弱势群体社会保障和服务。提高最低工资标准，健全企业职工工资正常增长机制。扎实推进扶贫攻坚，抓好下山移民，发展山区经济，完善生态补偿机制，支持金磐扶贫开发区转型发展。改善武义柳城、兰溪水亭等民族乡镇生产生活条件。完善困难群众价格补贴机制。

落实优抚安置政策，深化双拥工作，抓好国防动员和后备力量建设，推进民防建设。深入实施妇女儿童发展规划，加快发展老龄事业。积极做好侨务、对台、气象、统计、档案、修志、文物保护以及关心下一代等工作。认真完成第三次经济普查工作。积极做好援疆、援藏、援青及“双对口”工作。

（七）实施“实事惠民”行动，努力提高民生福祉

坚持量力而行、尽力而为的原则，围绕群众反映的突出问题，明确任务表、时间表、责任表，认真办好十方面民生实事。一是水环境治理方面。消灭垃圾河、黑河、臭河。实施城市供水水库水面保洁5万亩，农村河道综合整治471公里，河道保洁基本实现全覆盖。实施农村治污提速工程，行政村、自然村生活污水治理覆盖率分别提高到80%、60%以上，农户受益率提高到50%以上。加快污水处理厂技术改造，6家城镇生活污水处理厂排放水质达到国家一级A类标准，全市污水管网改造320公里。二是雾霾防治方面。全市5家燃煤电厂全部实施脱硫脱硝及除尘技术设备改造，淘汰黄标车1万辆。市区实施一环内煤改气，新增清洁能源城市公交车60辆以上。三是交通治堵方面。新增公交线路10条、优化32条，完成公交停靠站优化114个，新建公交首末站6个，新增公共自行车1万辆。四是美丽乡村建设方面。新建美丽乡村风景线9条，新创建市级美丽乡镇9个、精品村45个、秀美村90个。建设市级示范农村社区便民服务中心80个。完成农村改水32个村，覆盖人口1.9万人。五是食品监管方面。全部县（市、区）建成农产品安全快速检测室，并对公众开放。20家城镇农贸市场改造成为“放心市场”，三分之一城镇超市设立“放心柜”。六是低收入群体保障方面。最低工资标准提高8%以上，城乡居民最低生活保障标准提高8%以上，开工建设城镇保障性安居工程住房9000套，竣工4900套。七是就学就医方面。标准化中小学校比例达到50%，义务教育发展基本均衡县（市、区）通过省和国家评估验收，省等级幼儿园达到1000所。城乡居民健康体检115万人次，建立规范化健康档案450万份；市、县两级医院对口帮扶乡镇卫生院（社区卫生服务中心）52家；培训红十字救护员1万人次，普及培训4万人次。八是文化惠民方面。创建省、市级文化示范村（社区）30个，送戏下乡1000场，送电影下乡4.3万场。新增农村文化礼堂200家，开展非遗百村行活动，举办非遗精品演出100场；农村应急广播体系覆盖30%的村庄。九是就业和社会保障方面。新增城镇就业6万人。新增养老保险参保5万人、医疗保险参保2.5万人、工伤保险参保2万人、生育保险参保2万人、失业保险参保3万人。实现残疾人就业创业帮扶1000人。十是居家养老方面。新建城乡社区居家养老服务照料中心1200家以上，新增机构养老床位1000张以上。

（八）深化“正风提效”行动，切实转变政府职能

加快政府自身改革。加大简政放权力度，依法取消一批不符合全面深化改革要求的行政权力，继续下放一批市级审批权限。建立权力清单制度，规范权力运行。制定企业投资项目负面清单和市场准入标准。推进审批服务标准化和流程再造，建立“政务超市”，推行一表受理、并联审批、联合踏勘、多证联办制度。建设网上政务大厅。推进工商注册便利化，改先证后照为先照后证，改注册资本实缴登记制为认缴登记制。培育规范中介机构，建立公开公平的中介市场竞争机制。

优化政府机构设置和职能配置，完善行政运行机制。调整完善工商和食品药品监管体制，整合卫生、人口计生管理机构。坚持权责一致，深化基层行政执法体制改革，促进执法重心下移。深化各开发区、产业集聚区、风景旅游区体制改革。

严格执行中央、省委和市委关于改进工作作风、密切联系群众的各项规定，切实解决政府部门“门好进、脸好看、事难办”的问题。组织开展“千企万人双评议”活动，实施绩效管理和项目化考核，严格实行首问责任制和公务员问责制。健全调查研究制度，构建科学的政府决策咨询体系。推进政府和部门预算决算公开，提高财政资金使用绩效，市县两级政府“三公”经费预算支出削减30%。加强行政监察和审计监督，健全民主监督、法律监督、舆论监督机制，推进决策、管理、服务和结果公开。

各位代表，建设人民满意的服务型政府，是发展的要求、人民的期盼。我们将按照“照镜子、正衣冠、洗洗澡、治治病”的总要求，认真开展以“为民务实清廉”为主要内容的党的群众路线教育实践活动，不折不扣地完成各个重点环节的教育任务，确保在干部深受教育、突出问题得到解决、作风形象明显改进、人民群众真正满意等方面取得实效。认真落实市人大及其常委会各项决议决定，更加自觉接受人大依法监督和政协民主监督，主动接受司法监督、舆论监督和社会公众监督，积极听取人大代表、政协委员、民主党派、工商联、无党派人士、人民团体和各界人士的意见建议。推进科学决策、依法行政，以雷厉风行的状态、敢于担当的品德、一抓到底的韧劲、务实创新的方法，努力完成全年目标任务，不辜负全市人民的重托。

各位代表！今年改革发展的任务艰巨、责任重大。让我们在省委省政府和市委的正确领导下，解放思想，开拓进取，同舟共济，真抓实干，为加快“两富”现代化都市区建设而努力奋斗！
